# Supplementary material for: Noise-Reduction and Sensitivity-Enhancement of a Sleeping Beauty-Based Tet-On System
Source: Genes (Basel). 2022 Sep 20;13(10):1679. doi: 10.3390/genes13101679 (PMC9602432; doi:10.3390/genes13101679)
Supplement: Supplementary file 1 [file genes-13-01679-s001.zip › genes-1905640-supplementary.pdf]

Noise-Reduction and Sensitivity-Enhancement of a Sleeping Beauty-Based Tet-On System

Sarah C. Saunderson, SM Ali Hosseini-Rad and Alexander D. McLellan

MDPI GENES (In Review)

SUPPLEMENTARY DATA

Suppl. Figure S1. Schematic of the vector and gene orientation of the primary vector used in the study.

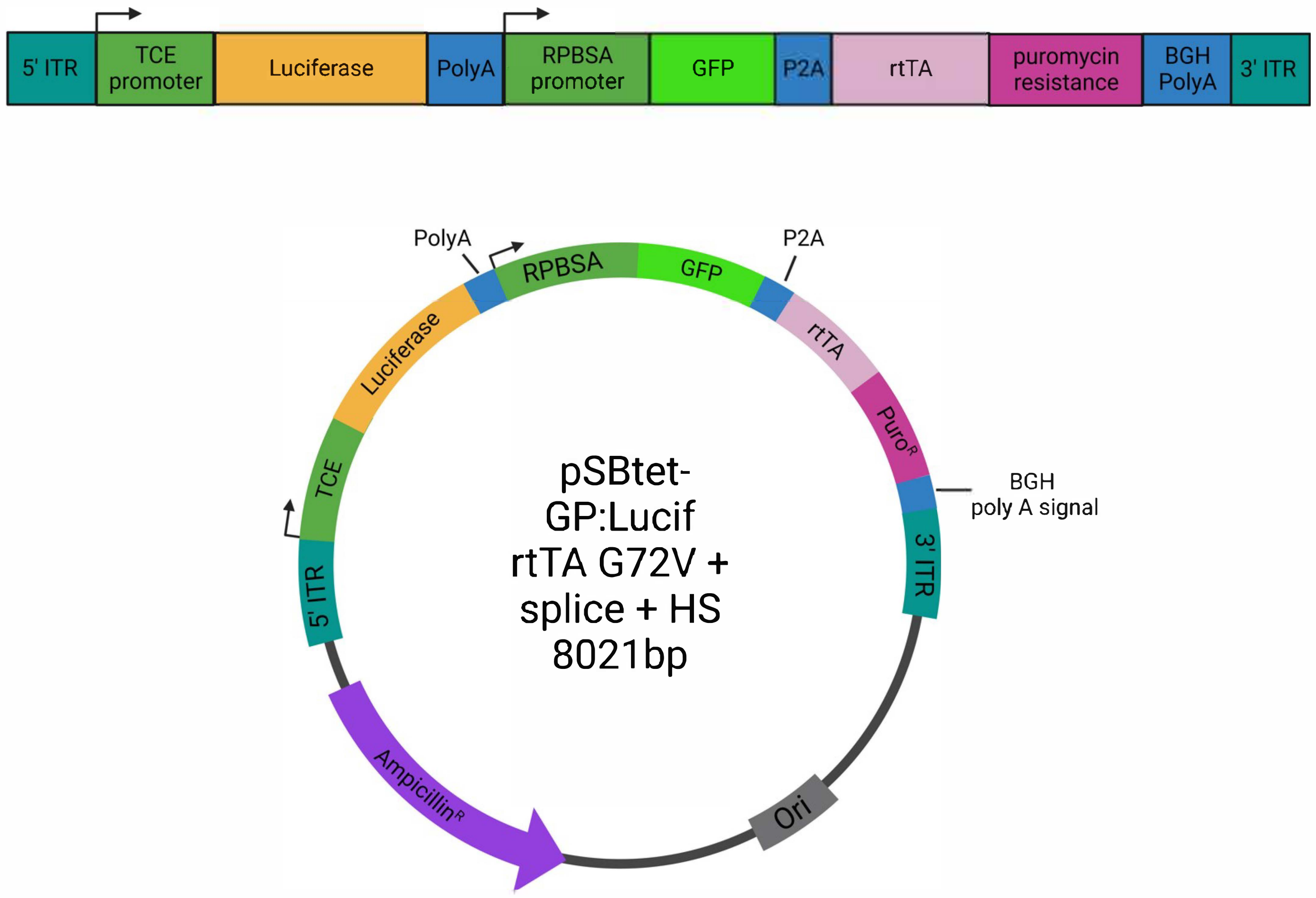

Figure S2. GFP expression of SB-transposed HEK293T cells.

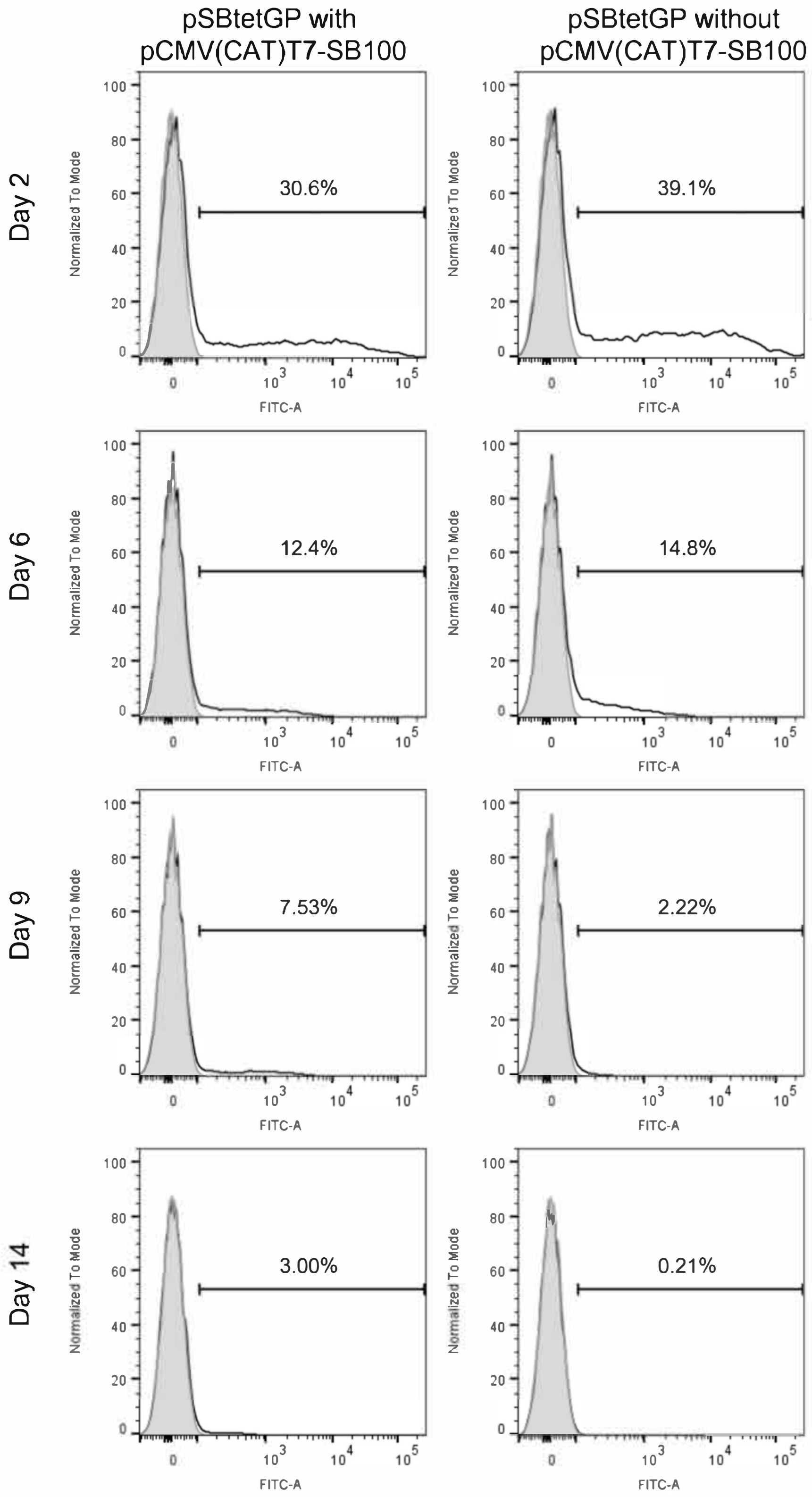

Suppl. Figure S2: Loss of transient expression of GFP encoded by the pSBTet vector with and without co-transfection with the pSB100X transposase vector. HEK293T cells were lipofectamine 3000 transfected with pSBtetGP:Luciferase plasmid with or without the pCMV(CAT)T7-SB100 transposase containing plasmid. HEK293T cells were analysed on days 2, 6, 9 and 14 for GFP expression by flow cytometry. Transfected cells (black line) or untransfected control cells (grey shaded peak). Results represent one pilot experiment.

Suppl. Table S1:

Position (bp)	splice site	Sequence	Score*	Intron GC*	Cryptic	Constit-utive	Confid-ence**
215 (G72)	cryptic acceptor	cccctggaagGC GAGTCATG	3.637	0.629	0.805	0.187	0.768
320	cryptic acceptor	cgcccaacagAG AAACAGTA	2.567	0.600	0.681	0.305	0.552
326	cryptic donor	CAGAGAAA CAgtacgaaacc	6.389	0.543	0.875	0.093	0.894
367	cryptic acceptor	cctgtgtcagCA AGGCTTCT	3.704	0.557	0.862	0.130	0.849
392	cryptic donor	AGAACGCA CTgtacgctctg	4.895	0.571	0.899	0.073	0.919
408	cryptic donor	TCTGTCCGC Cgtgggccact	5.697	0.500	0.676	0.264	0.609
456	cryptic donor	GGAGCATC AAgtagcaaaag	5.673	0.486	0.880	0.086	0.903
541	cryptic acceptor	cgaccggcagG GAGCCGAA C	5.154	0.529	0.870	0.124	0.858

Table S1. Prediction of cryptic acceptor/donor splice sites within original rtTA. Criteria: Acceptor site cutoff: 2.2 / Donor site cutoff: 4.5. *Scores of the preprocessing models reflecting splice site strength, i.e. a PSSM for putative acceptor sites, and an MDD model for putative donor sites. Intron GC values correspond to 70 nt of the neighboring intron. **Activations are output values of the backpropagation networks used for classification. High values for one class with low values of the other class imply a good classification. Confidence is a measure expressing the differences between output activations and range between zero (undecided) to one (perfect classification). All mutations (except 215 / G72V) were synonymous.

Suppl. Table S2: Percent of viable HEK293T following selection with puromycin from the same experiment as detailed in Figure 1B.

Transfection	Puromycin	Viability
**Untransfected**	**0 µg/mL**	**87.5%**
**Untransfected**	**2 µg/mL**	**1.6%**
**pSBtetGP with pCMV(CAT)T7-SB100**	**2 µg/mL**	**89.4%**
**pSBtetGP without pCMV(CAT)T7-SB100**	**2 µg/mL**	**1.2%**

Supplementary Methods

Sequences of rtTA-M2 variants used in the study. Changed nucleotides relative to the original rtTA-M2 are highlighted.

Original rtTA-M2

ATGTCTAGACTGGACAAGAGCAAAGTCATAAACGGCGCTCTGGAATTACTCAATGGAGTCGGTATCGAAGGCCTGACGACAAG
GAAACTCGCTCAAAAGCTGGGAGTTGAGCAGCCTACCCTGTACTGGCACGTGAAGAACAAGCGGGCCCTGCTCGATGCCCTGCC

AATCGAGATGCTGGACAGGCATCATACCCACTTCTGCCCCCTGGAAGGCGAGTCATGGCAAGACTTTCTGCGGAACAACGCCAA
GTCATTCCGCTGTGCTCTCCTCTCACATCGCGACGGGGCTAAAGTGCATCTCGGCACCCGCCCAACAGAGAAACAGTACGAAACC
CTGGAAAATCAGCTCGCGTTCCTGTGTCAGCAAGGCTTCTCCCTGGAGAACGCACTGTACGCTCTGTCCGCCGTGGGCCACTTTA
CACTGGGCTGCGTATTGGAGGAACAGGAGCATCAAGTAGCAAAAGAGGAAAGAGAGACACCTACCACCGATTCTATGCCCCCA
CTTCTGAGACAAGCAATTGAGCTGTTCGACCGGCAGGGAGCCGAACCTGCCTTCCTTTTCGGCCTGGAACTAATCATATGTGGCC
TGGAGAAACAGCTAAAGTGCGAAAGCGGCGGGCCGGCCGACGCCCTTGACGATTTTGACTTAGACATGCTCCCAGCCGATGCCC
TTGACGACTTTGACCTTGATATGCTGCCTGCTGACGCTCTTGACGATTTTGACCTTGACATGCTCCCCGGG

rtTA-M2 G72V

ATGTCTAGACTGGACAAGAGCAAAGTCATAAACGGCGCTCTGGAATTACTCAATGGAGTCGGTATCGAAGGCCTGACGACAAG
GAAACTCGCTCAAAAGCTGGGAGTTGAGCAGCCTACCCTGTACTGGCACGTGAAGAACAAGCGGGCCCTGCTCGATGCCCTGCC
AATCGAGATGCTGGACAGGCATCATACCCACTTCTGCCCCCTGGAAGTGGAGTCATGGCAAGACTTTCTGCGGAACAACGCCAA
GTCATTCCGCTGTGCTCTCCTCTCACATCGCGACGGGGCTAAAGTGCATCTCGGCACCCGCCCAACAGAGAAACAGTACGAAACC
CTGGAAAATCAGCTCGCGTTCCTGTGTCAGCAAGGCTTCTCCCTGGAGAACGCACTGTACGCTCTGTCCGCCGTGGGCCACTTTA
CACTGGGCTGCGTATTGGAGGAACAGGAGCATCAAGTAGCAAAAGAGGAAAGAGAGACACCTACCACCGATTCTATGCCCCCA
CTTCTGAGACAAGCAATTGAGCTGTTCGACCGGCAGGGAGCCGAACCTGCCTTCCTTTTCGGCCTGGAACTAATCATATGTGGCC
TGGAGAAACAGCTAAAGTGCGAAAGCGGCGGGCCGGCCGACGCCCTTGACGATTTTGACTTAGACATGCTCCCAGCCGATGCCC
TTGACGACTTTGACCTTGATATGCTGCCTGCTGACGCTCTTGACGATTTTGACCTTGACATGCTCCCCGGG

rtTA-M2 SE

ATGTCTAGACTGGACAAGAGCAAAATCATAAACGGCGCTCTGGAATTACTCAATGGAGTCGGTATCGAAGGCCTGACGACAAGG
AAACTCGCTCAAAAGCTGGGAGTTGAGCAGCCTACCCTGTACTGGCACGTGAAGAACAAGCGGGCCCTGCTCGATGCCCTGCCA
ATCGAGATGCTGGACAGGCATCATACCCACTCCTGCCCCCTGGAAGGCGAGTCATGGCAAGACTTTCTGCGGAACAACGCCAAG
TCATACCGCTGTGCTCTCCTCTCACATCGCGACGGGGCTAAAGTGCATCTCGGCACCCGCCCAACAGAGAAACAGTACGAAACCC
TGGAAAATCAGCTCGCGTTCCTGTGTCAGCAAGGCTTCTCCCTGGAGAACGCACTGTACGCTCTGTCCGCCGTGGGCCACTTTAC
ACTGGGCTGCGTATTGGAGGAACAGGAGCATCAAGTAGCAAAAGAGGAAAGAGAGACACCTACCACCGATTCTATGCCCCCAC
TTCTGAAACAAGCAATTGAGCTGTTCGACCGGCAGGGAGCCGAACCTGCCTTCCTTTTCGGCCTGGAACTAATCATATGTGGCCT
GGAGAAACAGCTAAAGTGCGAAAGCGGCGGGCCGGCCGACGCCCTTGACGATTTTGACTTAGACATGCTCCCAGCCGATGCCCT
TGACGACTTTGACCTTGATATGCTGCCTGCTGACGCTCTTGACGATTTTGACCTTGACATGCTCCCCGGG

One cryptic splice site remaining at nt 215

Position (bp)	Putative splice site	Sequence	Score*	Intron GC*	Alt./Cryptic	Constitutive	Confidence**
215<br>(G72V)	cryptic acceptor	cccctggaagGCGAGTCATG	3.637	0.629	0.805	0.187	0.768

rtTA-M2 ΔSpl

ATGTCTAGACTGGACAAGAGCAAAGTCATAAACGGCGCTCTGGAATTACTCAATGGAGTCGGTATCGAAGGCCTGACGACAAG
GAAACTCGCTCAAAAGCTGGGAGTTGAGCAGCCTACCCTGTACTGGCACGTGAAGAACAAGCGGGCCCTGCTCGATGCCCTGCC
AATCGAGATGCTGGACAGGCATCATACCCACTTCTGCCCCCTGGAAGGCGAGTCATGGCAAGACTTTCTGCGGAACAACGCCAA
GTCATTCCGCTGTGCTCTCCTCTCACATCGCGACGGGGCTAAAGTGCATCTCGGCACCCGCCCAACCGAGAAACAATACGAAACC
CTGGAAAATCAGCTCGCGTTCCTGTGTCAACAAGGCTTCTCCCTGGAGAACGCACTTTACGCTCTGTCCGCCGTTGGCCACTTTAC
ACTGGGCTGCGTATTGGAGGAACAGGAGCATCAAGTTGCAAAAGAGGAAAGAGAGACACCTACCACCGATTCTATGCCCCCACT
TCTGAGACAAGCAATTGAGCTGTTCGACCGGCAAGGAGCCGAACCTGCCTTCCTTTTCGGCCTGGAACTAATCATATGTGGCCTG
GAGAAACAGCTAAAGTGCGAAAGCGGCGGGCCGGCCGACGCCCTTGACGATTTTGACTTAGACATGCTCCCAGCCGATGCCCTT
GACGACTTTGACCTTGATATGCTGCCTGCTGACGCTCTTGACGATTTTGACCTTGACATGCTCCCCGGG

rtTA-M2 G72V + SE

ATGTCTAGACTGGACAAGAGCAAAATCATAAACGGCGCTCTGGAATTACTCAATGGAGTCGGTATCGAAGGCCTGACGACAAGG
AAACTCGCTCAAAAGCTGGGAGTTGAGCAGCCTACCCTGTACTGGCACGTGAAGAACAAGCGGGCCCTGCTCGATGCCCTGCCA
ATCGAGATGCTGGACAGGCATCATACCCACTCCTGCCCCCTGGAAGTGGAGTCATGGCAAGACTTTCTGCGGAACAACGCCAAG
TCATACCGCTGTGCTCTCCTCTCACATCGCGACGGGGCTAAAGTGCATCTCGGCACCCGCCCAACAGAGAAACAGTACGAAACCC
TGGAAAATCAGCTCGCGTTCCTGTGTCAGCAAGGCTTCTCCCTGGAGAACGCACTGTACGCTCTGTCCGCCGTGGGCCACTTTAC
ACTGGGCTGCGTATTGGAGGAACAGGAGCATCAAGTAGCAAAAGAGGAAAGAGAGACACCTACCACCGATTCTATGCCCCCAC
TTCTGAAACAAGCAATTGAGCTGTTCGACCGGCAGGGAGCCGAACCTGCCTTCCTTTTCGGCCTGGAACTAATCATATGTGGCCT
GGAGAAACAGCTAAAGTGCGAAAGCGGCGGGCCGGCCGACGCCCTTGACGATTTTGACTTAGACATGCTCCCAGCCGATGCCCT
TGACGACTTTGACCTTGATATGCTGCCTGCTGACGCTCTTGACGATTTTGACCTTGACATGCTCCCCGGG

rtTA-M2 G72V + ΔSpl

ATGTCTAGACTGGACAAGAGCAAAGTCATAAACGGCGCTCTGGAATTACTCAATGGAGTCGGTATCGAAGGCCTGACGACAAG
GAAACTCGCTCAAAAGCTGGGAGTTGAGCAGCCTACCCTGTACTGGCACGTGAAGAACAAGCGGGCCCTGCTCGATGCCCTGCC
AATCGAGATGCTGGACAGGCATCATACCCACTTCTGCCCCCTGGAAGTGGAGTCATGGCAAGACTTTCTGCGGAACAACGCCAA
GTCATTCCGCTGTGCTCTCCTCTCACATCGCGACGGGGCTAAAGTGCATCTCGGCACCCGCCCAACCGAGAAACAATACGAAACC
CTGGAAAATCAGCTCGCGTTCCTGTGTCAACAAGGCTTCTCCCTGGAGAACGCACTTTACGCTCTGTCCGCCGTTGGCCACTTTAC
ACTGGGCTGCGTATTGGAGGAACAGGAGCATCAAGTTGCAAAAGAGGAAAGAGAGACACCTACCACCGATTCTATGCCCCCACT
TCTGAGACAAGCAATTGAGCTGTTCGACCGGCAAGGAGCCGAACCTGCCTTCCTTTTCGGCCTGGAACTAATCATATGTGGCCTG
GAGAAACAGCTAAAGTGCGAAAGCGGCGGGCCGGCCGACGCCCTTGACGATTTTGACTTAGACATGCTCCCAGCCGATGCCCTT
GACGACTTTGACCTTGATATGCTGCCTGCTGACGCTCTTGACGATTTTGACCTTGACATGCTCCCCGGG

rtTA-M2 G72V + SE + ΔSpl

ATGTCTAGACTGGACAAGAGCAAAATCATAAACGGCGCTCTGGAATTACTCAATGGAGTCGGTATCGAAGGCCTGACGACAAGG
AAACTCGCTCAAAAGCTGGGAGTTGAGCAGCCTACCCTGTACTGGCACGTGAAGAACAAGCGGGCCCTGCTCGATGCCCTGCCA
ATCGAGATGCTGGACAGGCATCATACCCACTCCTGCCCCCTGGAAGTGGAGTCATGGCAAGACTTTCTGCGGAACAACGCCAAG
TCATACCGCTGTGCTCTCCTCTCACATCGCGACGGGGCTAAAGTGCATCTCGGCACCCGCCCAACCGAGAAACAATACGAAACCC
TGGAAAATCAGCTCGCGTTCCTGTGTCAACAAGGCTTCTCCCTGGAGAACGCACTTTACGCTCTGTCCGCCGTTGGCCACTTTACA
CTGGGCTGCGTATTGGAGGAACAGGAGCATCAAGTTGCAAAAGAGGAAAGAGAGACACCTACCACCGATTCTATGCCCCCACTT
CTGAAACAAGCAATTGAGCTGTTCGACCGGCAAGGAGCCGAACCTGCCTTCCTTTTCGGCCTGGAACTAATCATATGTGGCCTGG
AGAAACAGCTAAAGTGCGAAAGCGGCGGGCCGGCCGACGCCCTTGACGATTTTGACTTAGACATGCTCCCAGCCGATGCCCTTG
ACGACTTTGACCTTGATATGCTGCCTGCTGACGCTCTTGACGATTTTGACCTTGACATGCTCCCCGGG

No cryptic splice sites in rtTA-M2 G72V + SE + ΔSpl predicted after mutations.

Position (bp)	Putative splice site	Sequence	Score*	Intron GC*	Alt./Cryptic	Constitutive	Confidence**
	not detected						

Mutations were introduced for each splice site (Superscript numbers indicate the position of that nucleotide within rtTA).

215:

^{214} GCC ^{216} (G72) → GTG (V72)

320:

^{316} ACA ^{318} (T106) → ACC (T106)

326:

^{325} CAG ^{327} (Q109) → CAA (Q109)

367:

^{364} CAG ^{366} (Q122) → CAA (Q122)

392:

^{391} CTG ^{393} (L131) → CTT (L131)

408:

^{409} GTG ^{411} (V137) → GTT (V137)

456:

^{457} GTA ^{459} (V153) → GTT (V153)

541:

^{538} CAG ^{540} (Q180) → CAA (Q180)
